# Microbiota as key factors in inflammatory bowel disease

**DOI:** 10.3389/fmicb.2023.1155388

**Published:** 2023-10-13

**Authors:** Zachary White, Ivan Cabrera, Isabel Kapustka, Teruyuki Sano

**Affiliations:** Department of Microbiology and Immunology, College of Medicine, University of Illinois at Chicago, Chicago, IL, United States

**Keywords:** host-pathogen interaction, gut innate immunity, IELs, ILCs, protective bacteria for IBD

## Abstract

Inflammatory Bowel Disease (IBD) is characterized by prolonged inflammation of the gastrointestinal tract, which is thought to occur due to dysregulation of the immune system allowing the host’s cells to attack the GI tract and cause chronic inflammation. IBD can be caused by numerous factors such as genetics, gut microbiota, and environmental influences. In recent years, emphasis on commensal bacteria as a critical player in IBD has been at the forefront of new research. Each individual harbors a unique bacterial community that is influenced by diet, environment, and sanitary conditions. Importantly, it has been shown that there is a complex relationship among the microbiome, activation of the immune system, and autoimmune disorders. Studies have shown that not only does the microbiome possess pathogenic roles in the progression of IBD, but it can also play a protective role in mediating tissue damage. Therefore, to improve current IBD treatments, understanding not only the role of harmful bacteria but also the beneficial bacteria could lead to attractive new drug targets. Due to the considerable diversity of the microbiome, it has been challenging to characterize how particular microorganisms interact with the host and other microbiota. Fortunately, with the emergence of next-generation sequencing and the increased prevalence of germ-free animal models there has been significant advancement in microbiome studies. By utilizing human IBD studies and IBD mouse models focused on intraepithelial lymphocytes and innate lymphoid cells, this review will explore the multifaceted roles the microbiota plays in influencing the immune system in IBD.

## Introduction

Inflammatory bowel disease (IBD) is characterized as a chronic immune-mediated inflammatory disease affecting the gastrointestinal tract. IBD can be divided into two subgroups: Crohn’s Disease (CD), and Ulcerative Colitis (UC) (Crohn’s and Colitis Foundation, 2014). CD can occur anywhere along the gastrointestinal tract and involve patches of healthy tissue mixed between inflamed areas, on the other hand, UC is limited to the colon and has continuous inflammation of large areas of the colon (Fakhoury et al., 2014). The National Institute of Allergy and Infectious Diseases (NIAID) is focusing on IBD as one of the specific autoimmune diseases for intense study.^1^ The prevalence of IBD is increasing worldwide, recent reports have recorded that an estimated 2 million people in North America, 3.2 million people in Europe, and millions more in east and southern Asia, have been diagnosed with IBD as of 2020 (Ananthakrishnan et al., 2020; Olfatifar et al., 2021; Zhao et al., 2021). With rates of IBD rising all over the world, development of new strategies to combat IBD are critically needed. Although the underlying causes of IBD are ill-defined, IBD is thought to develop due to microbial, environmental, genetic, and immune-mediated factors (Khan et al., 2019; Glassner et al., 2020; Mentella et al., 2020). Recently, the importance of the microbiome in the development of IBD has been at the forefront of study (Khan et al., 2019; Somineni and Kugathasan, 2019).

**Figure 1:**
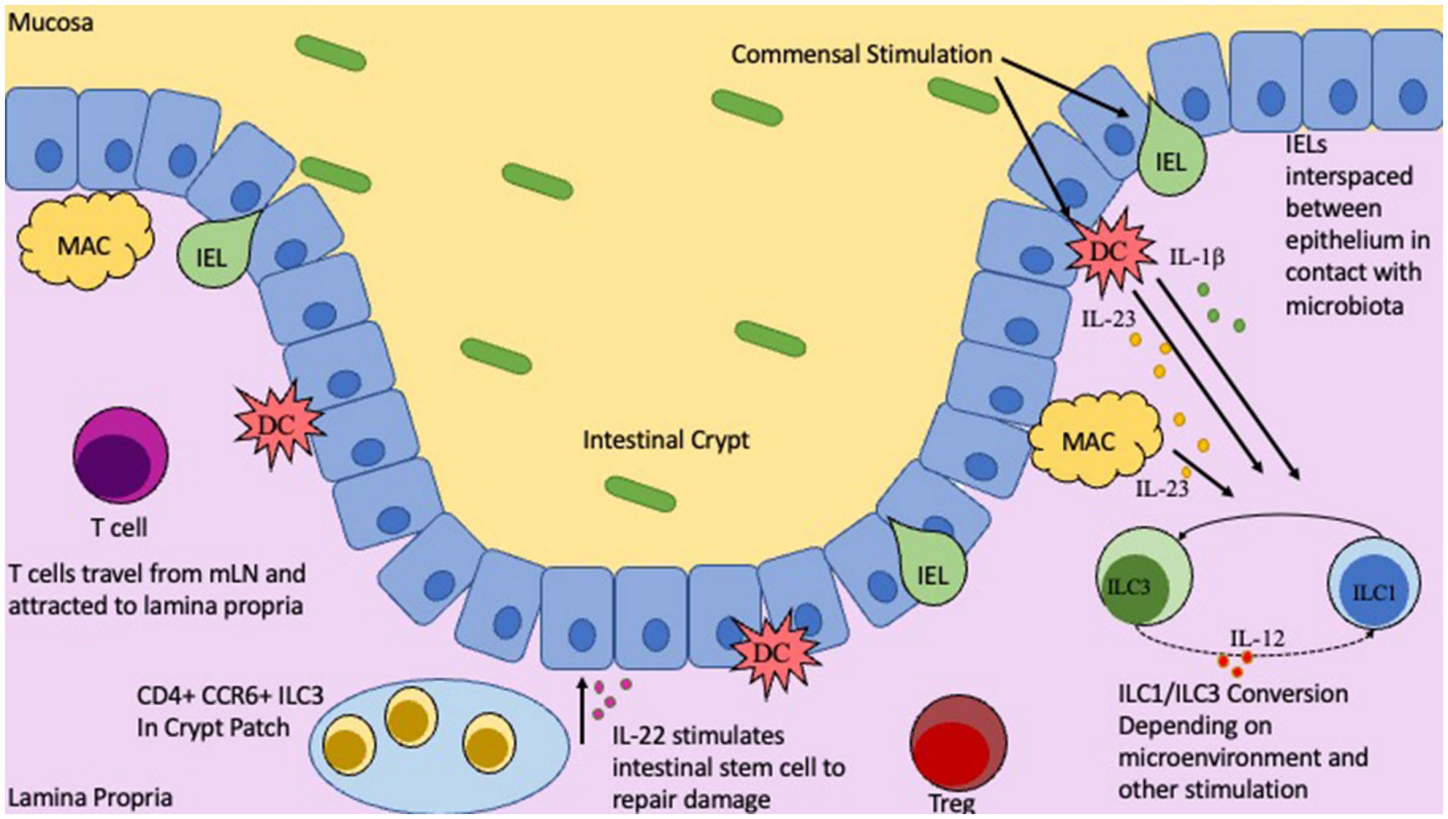
Location and functions of T cells, IELs, and ILCs in the Gut. Intestinal epithelial barrier with gut mucosa and Lamina Propria on either side. Commensal microbiota in the gut lumen will stimulate innate immune cells, DCs and Macrophages, as well as IELs. Depending on nature of stimulation and by which commensal will influence IEL cytokine production, along with IL-23 and IL-1b from DCs/Macs to trigger ILC1/ILC3 conversion. CD4+ CCR6+ ILC3s located in the crypt patch will produce IL-22 to stimulate repair and regeneration mechanisms.

Our microbiomes contain more than 100 trillion different microorganisms, including bacteria, viruses, fungi, and protozoa. The gastrointestinal (GI) tract is one niche that harbors significant populations of these microorganisms (Figure 1). The predominant bacterial populations in a healthy microbiome are *Firmicutes*, *Actinobacteria*, *Bacteroidetes*, and *Verrucomicrobia* (Pickard et al., 2017; Kho and Lal, 2018). Importantly, the distribution of bacterial species throughout the GI tract varies (Table 1). The colon harbors the greatest number and diversity of bacteria with the highest proportion of colon colonizing bacteria being *Bacteroides*, *Bifidobacterium,* and *Clostridiales* (Vuik et al., 2019). These bacteria can survive in this location because they might be adept at using enzymes to break down and digest complex polysaccharides which are indigestible by the host (Flint et al., 2012). The stomach on the other hand contains comparatively few microorganisms due to its high-stress conditions, with its main populations being *Streptococcus*, *Prevotella*, *Rothia*, and *Veillonella* (Nardone and Compare, 2015). Moreover, the small intestine also contains lower bacterial populations compared to colon, most likely due to its proximity to the stomach, and is rich in mono- and di-saccharides which promote *Proteobacteria* and *Firmicutes* (Vuik et al., 2019; Leite et al., 2020). This description of the “healthy” microbiome is not complete however, as it is noted that during dysbiosis there is the possibility that these normally commensal bacteria can emerge as pathobionts due to the altered environment of the gut. Therefore, a comprehensive analysis of potential pathobionts and opportunistic microbes is required before we can define a truly “healthy” microbiome (Chervy et al., 2020; Mancini et al., 2021). The gut microbiome can play a critical role in gut homeostasis, metabolism, immune activation, and defending against intestinal pathogens. Thus, the study of how the gut microbiome influences the immune system to either combat or promote IBD will provide valuable insights for new drug targets or therapeutics.

**Table 1:** Distinct microbiota associations.

Tissue	Phyla: Genus	Environment	Reference
Stomach	Firmicutes: *Lactobacillus*, *Streptococcus*, *Enterococcus*, Veillonella Proteobacteria: *Helicobacter*, Haemaphilus, Neisseria Actinobacteria: Rothia, Propionibacterium Bacteroidetes: Bacteroides Prevotella	Partially Aerobic Highly acidic	Nardone and Compare (2015) Yang et al. (2013) Wu et al. (2014) Martinez-Guryn et al. (2019)
Duodenum	Firmicutes: *Lactobacillus*, *Clostridium*, *Staphylococcus*, *Streptococcus* Bacteroidetes: Bacteroides Actinobacteria: *Bifidobacterium* Proteobacteria: Neisseria	Partially Aerobic Neutral	Wang et al. (2005) Cheng et al. (2013) Kastl et al. (2020) Martinez-Guryn et al. (2019)
Jejunum	Firmicutes: *Streptococcus*, *Veillonella*, *Clostridium*, *Enterococcus*, *Lactobacillus* Proteobacteria: *Escherichia*, *Haemophilus*, *Neisseria*, *Klebsiella*, *Citrobacter* Bacteroidetes: *Prevotella*, *Bacteroides* Actinobacteria: *Bifidobacterium*, *Rothia*, *Actinomyces* Fusobacteria: *Fusobacterium*, *Leptotrichia*	Partially Aerobic Neutral	Sundin et al. (2017) Martinez-Guryn et al. (2019)
Ileum	Firmicutes: Clostridium, Peptostreptococcus, Lactobacilli, *Enterococcus* Actinobacteria: *Bifidobacterium* Proteobacteria: *Heliobacter*, *Escherichia* Bacteroidetes: Bacteroides, Prevotella	Partially Aerobic Acidic	Villmones et al. (2018) Martinez-Guryn et al. (2019)
Colon	Firmicutes: *Clostriudm*, *Ruminococcus*, Lactobacilli Proteobacteria: *Escherichia*, *Enterobacter*, *Heliobacter*, *Klebsiella* Bacteroidetes: Bacteroidesm Orevotella, Alistipes Actinobacteria: *Bifidobacterium* Verrucomicrobia: Akkermansia	Highly Anaerobic Neutral	Mailhe et al. (2018) Thursby and Juge (2017) Martinez-Guryn et al. (2019)

Associations of microbiota and regions of GI tract. There are specific bacteria that tend to colonize in distinct locations in the stomach and intestines. This is most likely due to the local micro-environment that promotes certain bacteria over others.

## The microbiome and inflammatory bowel disease

The gut microbiome contains high concentrations of commensals, such that immunomodulatory bacteria will constantly be providing stimuli to the host’s immune cells, especially dendritic cells and macrophages which extend their dendrites to capture such bacteria. During non-perturbed circumstances, this stimulation will lead to immunologic tolerance which prevents unwanted intestinal inflammation. It is believed that genetic susceptibility, environmental factors, and certain microbes can cause this controlled tolerance to become dysregulated and lead to the development of colitis. Although the mechanisms underlying how and why this occurs are not completely understood, many studies have indicated that the mere presence of bacteria significantly influences these processes. Studies have shown that genetically susceptible rodents that have been housed under germ-free (GF) conditions, show little to no intestinal inflammation. Whereas mice breed under specific pathogen free (SPF) conditions show symptoms of colitis (Sartor, 1997; Sellon et al., 1998; Keubler et al., 2015). Accordingly, mice that have been genetically targeted to delete the gene responsible for IL-10, a potent anti-inflammatory cytokine, will develop inflammation in the cecum and colon spontaneously, and had increased highly activated CD4+ T cells when housed in SPF conditions (Sellon et al., 1998; Keubler et al., 2015). Interestingly, IL-10 KO GF mice on the other hand had a lack of colitis and immune activation (Sellon et al., 1998). This induction of colitis in genetically susceptible mice has long been attributed to the human enterohepatic *Helicobacter Helicobacter hepaticus* (*H. hepaticus*) (Danne et al., 2017; Zhu et al., 2021). However, there is conflicting evidence to suggest that *H. hepatoicus* itself is not sufficient to induce colitis unless it is co-colonized with *Lactobacillis reuteri* (Dieleman et al., 2000; Whary et al., 2011). Additionally, fecal transplants from IBD human donors are sufficient to induce IBD symptoms in GF mice and that fecal transplantation from healthy donors does not induce these symptoms (Erben et al., 2014; Weingarden and Vaughn, 2017; Jang et al., 2021). This is thought to occur by the IBD donor microbiota preferentially stimulating Th17 and Th2 T cells over Tregs, while healthy microbiota induce more Tregs (Longman et al., 2013; Britton et al., 2019). Fittingly, it has been observed that when naïve CD4+ T cells were transferred from healthy mice into immunocompromised mice, colitis was induced (Powrie et al., 1994; Kullberg et al., 2006; Ostanin et al., 2009). The degree to which these mice were susceptible to colitis was directly associated with the composition of the mouse’s unique gut microbiome (Reinoso Webb et al., 2018), and this naive T cell transfer colitis model is widely used to study the effects of the microbiome on IBD pathogenesis (Ostanin et al., 2009).

Interestingly, it has been found that there are distinct actions that the hosts immune system with partake in response to colonization of the gut microbiota. For example, in 2014, it was observed that specific intestinal microbiota are preferentially coated with high levels of immunoglobulin A (IgA) and that the specific microbes that have this high IgA coating are found to dramatically increase susceptibility to colitis (Gaboriau-Routhiau et al., 2009; Palm et al., 2014). Utilizing this differential coating of IgA could be a powerful tool in distinguishing between potentially pathogenic bacteria and healthy commensals (Pabst and Slack, 2020). In humans, it has been demonstrated that IBD severity is closely associated with disruptions in the normal microbiota, known as dysbiosis (Schaubeck et al., 2016). Not only is the diversity of bacteria in healthy intestinal tissue much greater than that of inflamed intestinal tissue, but there is a marked change in the predominant bacteria colonized. It has been observed that patients with IBD are observed to have a reduction in Firmicutes and an increase in Proteobacteria, and a few members of Bacteroidetes (Sepehri et al., 2007; Alam et al., 2020; Lee and Chang, 2021). This change in composition and diversity is thought to lead to an impairment of several key immunomodulatory functions and reduction in intestinal barrier integrity thus allowing inflammation to occur and stimulate unnecessary immune responses (Lee and Chang, 2021). Importantly, in addition to diversity and composition of the microbiome being reduced an/or altered it has also been observed that the metagenomic, metatransciptomic, and metabolomic profiles of the microbiomes in IBD patients are altered (Lloyd-Price et al., 2019). For example, the measured metabolites produced by the microbiota in IBD patients are significantly less diverse than those without IBD. Additionally, they observe that Short Chain Fatty Acids (SCFAs) produced by the microbiota are reduced in patients with dysbiosis; specifically, the SCFA butyrate is consistently noted as being less abundant in dysbiosis. Lloyd-Price et al., 2019, hypothesize that this loss of butyrate is due to the depletion of important metabolite producers like *F. prausnitzii* and *R. hominis* (Lloyd-Price et al., 2019). Along these lines, this same group also observed that the primary bile acid cholate and its conjugates are consistently measured higher in dysbiosis from patients with CD, and conversely that the secondary bile acids deoxycholate and lithocholate are reduced in this condition (Duboc et al., 2013; Lloyd-Price et al., 2019). They conclude that the microbiota change in IBD-associated dysbiosis causes alterations in intestinal bile acid production which may lead to the inhibition of anti-inflammatory effects seen in some of the secreted bile acids resulting in chronic inflammation that is a hallmark of IBD (Duboc et al., 2013). Lastly, it was noted that IBD specific signals are significantly detectable at the RNA level in dysbiosis, this includes pathways known to be upregulated in IBD and known bacterial species that are observed to have different expression profiles in IBD patients (Schirmer et al., 2018). Adding to this complexity, recent studies showed that dysbiosis of oral microbiome in patients with periodontitis can also directly influence the development of gut inflammation by promoting harmful pathobiont colonization and supplying pathogenic T cells to the gut (Atarashi et al., 2017; Kitamoto et al., 2020; Kitamoto and Kamada, 2022). Microbiomes from other niches can influence microbiome-mediated gut tissue inflammation. However, treatment of IBD with antibiotics has shown conflicting outcomes; with some studies showing mild benefits and others with little to no effect (Nitzan et al., 2016; Abraham and Quigley, 2020). This may be explained by the variable locations of disease occurrence and the different mechanisms of action for the varying antibiotics tested. For example, if the treated antibiotics depleted more beneficial bacteria than pathogenic bacteria, the output of the treatment for IBD will be detrimental. So far, our knowledge cannot predict such outcomes because of the diversity of bacteria and their unknow immunomodulatory functions. Finally, the genetic indicators that are known to be associated with IBD are generally related to how the immune system interacts with the gut microbiota (Imhann et al., 2017; Aschard et al., 2019; Cohen et al., 2019). Thus, characterizing how commensal bacteria interact with the host’s tissues and immune system will be critical for combatting IBD. Taken together microbiome-dependent T cell activation and dysregulation can cause gut inflammation, while innate lymphoid cells (ILCs) and intra-epithelial lymphocytes (IELs), which are also abundant in the intestine and function similarly as T cells, likewise contribute to IBD. In this review, we will examine the contribution of T cells, ILCs and IELs in IBD pathogenesis.

## Microbiota influence on intra-epithelial lymphocytes

The microbiome has a significant impact on maturation and education of the immune system. To keep immune responses in check, commensal microbes induce immune tolerance to prevent unwanted immune activation and inflammation while still allowing for robust immune responses toward pathogens. This is a delicate process and any perturbations, such as dysbiosis or genetic susceptibility, may lead to improper immune responses and potentially IBD. One critical player are the intraepithelial lymphocytes (IELs). IELs are interspaced between epithelial cells in the intestinal barrier located underneath the mucosal layer. IELs are considered the first line of defense in the gastrointestinal tract as they are the first immune cell to encounter microbes colonizing the mucosa (Figure 1). Once IELs arrive at the intestinal barrier and position themselves between the epithelial cells, they do not return to circulation (Masopust et al., 2010; Van Kaer and Olivares-Villagómez, 2018). The distribution of IELs is not consistent along the entire gastrointestinal tract and differs between mice and humans (Camerini et al., 1993; Beagley et al., 1995; Tamura et al., 2003). The microbiome is seen as a potent modulator of IEL phenotypic composition and clonal expansion (Helgeland et al., 2004). It was observed that in GF rats there was a significant reduction in CD4+ IELs compared to conventionalized rats that showed notable populations of both CD4+ IELs as well as CD4/CD8α + double positive IELs (Helgeland et al., 2004; Harada et al., 2022). Interestingly, the GF rats displayed similar numbers of CD8+ IELs compared to conventionalized rats, indicating that while CD4 single and double positive IELs are highly influenced by the microbiota, CD8+ IELs seem to be relatively independent of microbiota (Bousso et al., 2000; Helgeland et al., 2004). Although it has been difficult to identify the different bacterial species in the microbiome that influence IEL composition, it has been noted that segmented filamentous bacteria (SFB) has the ability to significantly modulate IELs (Umesaki et al., 1999; Helgeland et al., 2004).

The majority of IELs express a CD8αα homodimer with around 90% of these expressing T cell receptors (TCRs) (Van Kaer and Olivares-Villagómez, 2018). These IELs can be divided into two groups, natural or induced IELs. Induced IELs form from naïve T cells that encounter antigens in the gut-associated lymphoid tissue (GALT) and then migrate to the intestine. Induced IELs can be further classified into CD4 or CD8αβ expressing. Induced IELs are typically thought to possess an effector-memory phenotype that aids in the defense against pathogens. Contrary to traditional thought there is some evidence to suggest that induced TCRαβ^+^ CD4^+^ IELs do not lose their CD8 chains such that they express both CD4 and CD8αα (Morrissey et al., 1995; Yap and Mariño, 2018). Studies have revealed that some commensals can modulate the generation and function of TCRαβ^+^ CD4^+^ CD8αα^+^ IELs (Zhou C. et al., 2019). They show that TCRαβ^+^ CD4^+^ IELs are absent in GF mice and that the addition of the commensal *Lactobacillus reuteri* can induce expansion of TCRαβ^+^ CD4^+^ IELs by downregulating ThPOK, a key transcriptional regulator of T cells (Cervantes-Barragan et al., 2017). Importantly, this specialized IEL subset has been associated with IBD and has been found to, upon stimulation by microbiota, induce alteration of Foxp3+ Treg into TCRαβ^+^ CD4^+^ CD8αα^+^ IELs during intestinal inflammation (Das et al., 2003; Cervantes-Barragan et al., 2017; Zhou C. et al., 2019). Moreover, a very recent report from the Kasper lab showed that dietary Fatty Acids (FAs) can be bio-transformed into Conjugated Linoleic Acids (CLAs) predominantly by the microbiota in the small intestine. They demonstrate that the CLAs then act as important modulators to maintain a healthy CD4^+^ CD8αα^+^ IEL pool, highlighting gut commensal’s role in preserving mucosal defenses (Song et al., 2023). The other subclass of induced IELs is TCRαβ^+^ CD8αβ^+^ IELs. These cells are derived from activated CD8^+^ T cells from the periphery and account for up to 15% of total IELs in mice and 80% in humans (Sujino et al., 2016). While physiological roles of these cells in intestine during homeostasis and pathogenesis are not fully understood yet, several studies have uncovered links between TCRαβ^+^ CD8αβ^+^ IELs and the microbiome. One study demonstrated a significant increase in TCRαβ^+^ CD8αβ^+^ IELs when GF mice were microbially colonized in SPF conditions (Jarry et al., 1990). Others have shown that reducing specific bacterial species with antibiotics can increase the number of TCRαβ^+^ CD8αβ^+^ IELs (Imaoka et al., 1996). Natural IELs on the other hand, will home directly to the intestinal epithelium after thymic development. Natural IELs include TCRγδ^+^, which are known to have high mobility throughout the intestinal epithelium (Chen B. et al., 2018). Interestingly, there is conflicting evidence for the role of TCRγδ^+^ IELs in colitis models. Some suggest that these cells act protectively against inflammatory damage to the epithelium; while others indicate that TCRγδ^+^ IELs act in a pathogenic fashion (Simpson et al., 1997; Canesso et al., 2017; Mariño et al., 2017; Nielsen et al., 2017). Some studies suggest that TCRγδ^+^ IELs help protect the intestinal epithelium from inflammatory damage by secreting TGF-β and KGF (Yang et al., 2004; Hu et al., 2018; Michaudel and Sokol, 2020). Although various papers indicated the relationships between microbiota and IELs up-regulation and activation, the repertoire of TCR on the IELs had been not investigated. Recently Bousbaine et al., reported that β-hexosaminidase, a conserved enzyme across commensals of the Bacteroidetes phylum, as a driver of CD4^+^ IEL differentiation. In addition, the paper nicely showed that the β-hexosaminidase-specific CD4^+^ IELs can play a protective role in a mouse model of colitis (Bousbaine et al., 2022). Utilizing and enhancing the protective mechanisms of our immune cells is a promising path for IBD therapeutics.

## Microbiota influence on innate lymphoid cells

**Figure 2:**
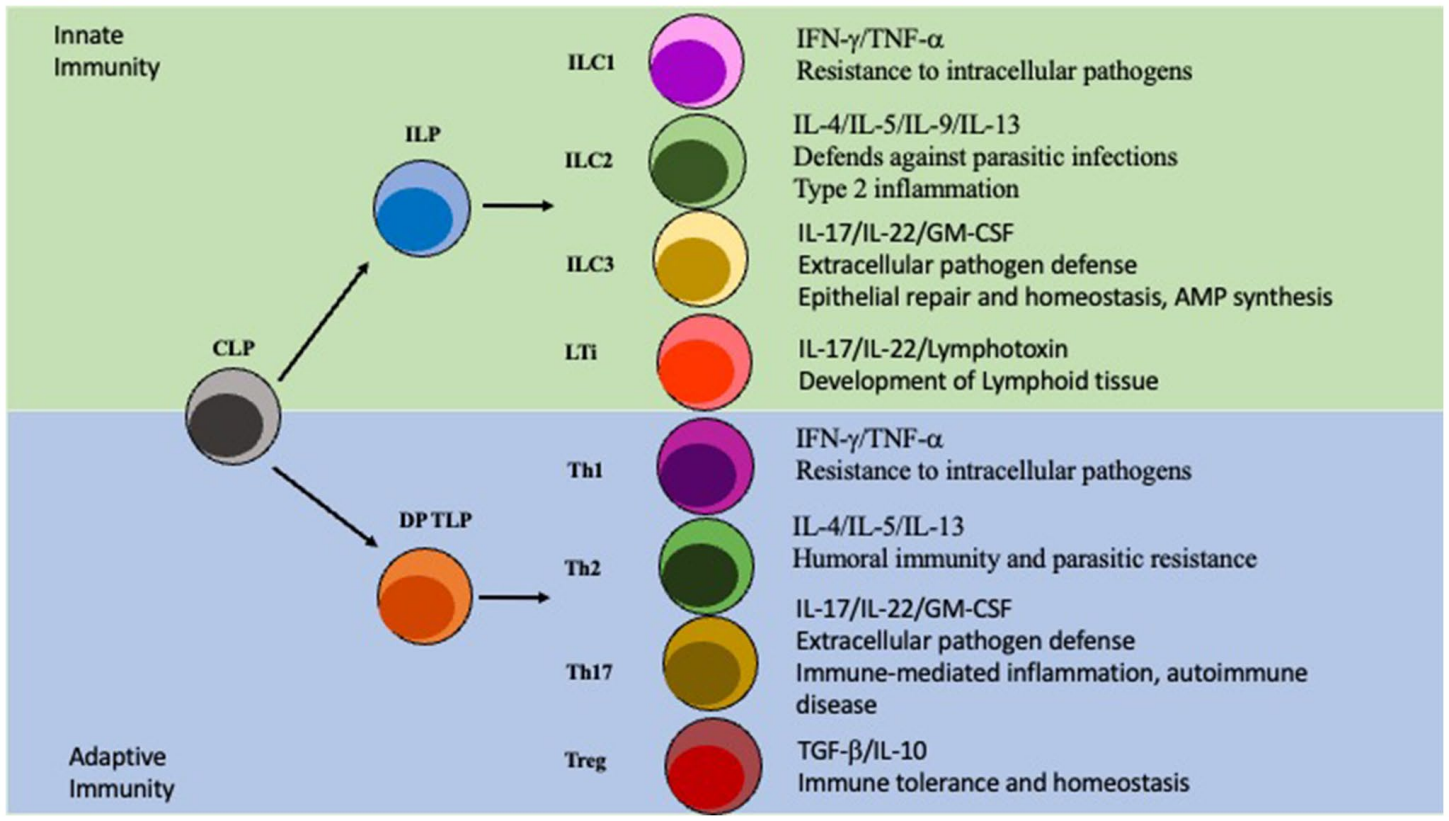
ILC *vs* T cell Differentiation. ILC and T cell differentiation. The Innate lymphoid progenitor (ILP) and the double positive T cell progenitor (DP TLP) arise from a common lymphoid progenitor (CLP). The ILP can then, depending on other signals, differentiate into ILC1/ILC2/ILC3/LTi cells which are part of the innate immunity. Similarly, the DP TLP can differentiate into a Th1/Th2/Th17/Treg identity and is part of the adaptive immunity.

Innate lymphoid cells (ILCs) are a class of immune cells that form from a common lymphoid progenitor (CLP) (Liu et al., 2011; Artis and Spits, 2015). ILCs are also associated with IBD pathogenesis by interacting directly and indirectly with the microbiota, regulating intestinal barrier integrity, and secreting cytokines. Dysfunction of ILCs will perturb gut homeostasis and can lead to gut inflammation and IBD. There are several subclasses of ILCs based on their phenotype: ILC1, ILC2, ILC3, NK/ LTi cells are usually included in descriptions of ILCs as they share many similarities in development and function (Liu et al., 2011; Panda and Colonna, 2019; Figure 2). ILCs are known to be primarily located at the mucosal surfaces. With the most well-known reservoir being the intestinal epithelial barrier, which allows extensive communication with the gut microbiota (Liu et al., 2011; Panda and Colonna, 2019). Although ILCs have been shown to modulate both innate and adaptive immunity through secretion of cytokines, they are considered a part of the innate immune system (Liu et al., 2011; Abt et al, 2015; Mortha and Burrows, 2018; Vivier et al., 2018). ILCs are seen as the innate immune systems counterpart to T cells because they produce similar effector cytokines but require no education by antigen presenting cells via TCR is required (Vivier et al., 2018; Panda and Colonna, 2019). ILCs are tissue-resident cells that are found in high numbers within the mucosa and have been associated with maintaining homeostasis, intestinal repair, and regeneration (Liu et al., 2011; Vivier et al., 2018). Importantly ILCs will constantly produce their effector cytokines at steady-state conditions and are not rapidly replenished by the circulation (Giuffrida et al., 2018; Luo et al., 2021). ILC1 cells are known to produce the key cytokines IFN-γ and TNF-α, as well as provide resistance to intracellular pathogens; with their adaptive counterparty being Th1 cells which is also regulated by the same master transcription factor T-bet (Fuchs, 2016; Vivier et al., 2018). ILC2 cells, whose T cell counterpart is Th2 and master transcription factor being GATA3, secrete several interleukins: IL-4, IL-5, IL-9, and IL-13. ILC2s are involved in fighting parasitic infections and in type two mediated inflammation (Vivier et al., 2018). Next, the ILC3s are regulated by the transcription factor RORγt and produce the effector cytokines IL-17, IL-22, and GM-CSF similarly to Th17 cells (Qiu et al., 2012; Vivier et al., 2018). They are involved in extracellular pathogen defense, epithelial repair, and homeostasis, along with stimulating AMP synthesis. LTi cells are important to the development of the secondary lymphoid organs, for example Peyer’s Patches on the small intestine (Sonnenberg et al., 2011; Van De Pavert, 2021). Although NK cells are also regulated by the transcription factor T-bet and produce IFNγ they are not directly differentiated into NK cells, instead arising from a separate, but related, NK progenitor that is reportedly governed by the expression of TOX, NFIL3, ID2, and ETS1 (Zhang and Huang, 2017; Vivier et al., 2018). Importantly, although the other ILCs are closely related to CD4 T cells NK cells on the other hand resemble CD8 cytotoxic T cells (Zhang and Huang, 2017).

In an IBD context, the balance between ILCs, particularly ILC1 and ILC3 is currently a major area of study, as this balance has been linked to intestinal inflammation (Chen L. et al., 2018; Castleman et al., 2020; Saez et al., 2021). However, ILC2s have recently been implicated in gut microbiome dependent inflammatory responses in the lung, indicating that ILC2s may also play a role in the development of inflammatory diseases but this is still unclear (Pu et al., 2021). ILC3s have been reported to perform several functions related to maintaining homeostasis in the gut. They can sense and communicate with the microbiota to influence intestinal epithelial cells to produce anti-microbial peptides, promote epithelial tissue repair and regeneration, and modulate the adaptive immune system (Buela et al., 2015; Diefenbach et al., 2020; Romera-Hernández et al., 2020; Zhou and Sonnenberg, 2020; Sepahi et al., 2021). The hallmark of ILC3 is the production of IL-17 and IL-22 and, in certain conditions, GM-CSF. One way the microbiome communicates with ILCs is through Short Chain Fatty Acids (SCFAs). For example, dietary fiber can be metabolized by the gut microbiome to produce SCFAs that in turn act as regulators for immune cells including ILC3s (Gasaly et al., 2021; Sepahi et al., 2021). Accordingly, the metabolites produced by commensals from dietary fibers regulate G-protein coupled receptors to promote optimal expansion of ILCs in the gut (Chun et al., 2019; Sonnenberg and Hepworth, 2019). It has been shown that metabolites produced by commensals will stimulate the AKT-STAT3 and ERK-STAT3 pathways to increase IL-22 production and ILC3 generation (Sarrabayrouse et al., 2014).

IL-22 is known to promote epithelial barrier integrity and encourage epithelial regeneration (Chun et al., 2019; Poholek et al., 2019). Its clinical relevance in IBD has been extensively documented. In short, IL-22 can induce mucosal repair and regeneration which is critical for treatment of IBD, and IL-23R, which is known to stimulate production of IL-22, is associated with the development of IBD (Geremia et al., 2011). Additionally, IL-22 is also demonstrated to stimulate the production of anti-microbial peptides (AMPs); specifically, the REGIII family (Chun et al., 2019; Willinger, 2019). ILC3s also communicate with the commensals by utilizing the ILC’s TLRs. TLR stimulation by commensal PAMPS can induce secretion of IL-22, IL-13, and IL-5 (Takatsu, 2011; Reece et al., 2014; Xuan et al., 2021). In conjunction with directly regulating ILCs, commensals can also indirectly influence ILCs by modulating myeloid and epithelial cells in the intestine. In mice, commensal microbes will stimulate intestinal resident myeloid cells to secrete IL-1β and IL-23. These two key cytokines in turn will stimulate ILC3s to produce the GM-CSF; GM-CSF can induce Treg generation and expansion via dendritic cells, enhancing tolerance (Pickert et al., 2009; Mortha et al., 2014; Mizoguchi et al., 2018; Miljković et al., 2021). Concurrently, IL-1β will also influence ILC3s to secrete IL-2 which also drives Treg generation and tolerance (Mizoguchi et al., 2018; Zhou L. et al., 2019). This occurs as Tregs will inhibit ILC3-associated colitis by preventing the secretion of IL-23 and IL-1β from tissue-resident macrophages that block the production of IL-22 (Saez et al., 2021).

Additionally, ILC3s are associated with the IBD susceptibility gene TNFSF15 as well as the adaptive immune response. The TNFSF15 gene encodes the protein TL1A which is the ligand for Death Receptor 3 (DR3) (Jostins et al., 2012; Castellanos et al., 2018). This ligand TL1A will act in concert with IL-23 and IL-1β to enhance IL-22 secretion and promote ILC3 expansion *ex vivo* (Longman et al., 2014; Ahn et al., 2015). Moreover, in mice colonized with Segmented Filamentous Bacteria (SFB) or IBD-associated microbiota such as Adherent-Invasive *E. coli* (AIEC), there is an increase in ILC3-produced IL-22 (Sano et al., 2015; Castellanos et al., 2018). It was revealed that this occurs by inducing TL1A expression on mononuclear phagocytes such that the increased expression of TL1A will lead to IL-22 secretion from ILC3s in a DR3 dependent manner. To confirm this association, ILC3 specific DR3 deletion in mice showed decreased IL-22 secretion from ILC3s and a higher susceptibility to chemical-induced colitis (Castellanos et al., 2018). Not only has it been observed that the microbiota can influence ILCs, but they also impact T cells in response to certain bacterial stimuli. For example, *E. coli* has been shown to induce Th17 mucosal immunity exacerbating colitis or arthritis severity (Viladomiu et al., 2017). In addition to commensals influencing ILCs, it has been demonstrated that cytokines produced by ILCs can regulate commensal bacteria; this is seen by T-bet+ type 1 ILCs (ILC1) that produce IFNγ and TNFα that can increase the permeability of the colonic epithelial cell line and thus influence the movement of commensals across the intestinal epithelium (Clark et al., 2005). This crosstalk between ILCs and commensals via myeloid cells is critical in maintaining homeostasis in colitis models. Expression of IFNγ in ILC1 was dependent on proper T-bet expression and that mice deficient in T-bet developed ILC-associated colitis due to an increase in IL-17 production (Powell et al., 2012). Furthermore, ILCs are directly associated with IBD. It has been noted that in IBD tissue samples there is a reduced number of NKp44^+^ ILC3s compared to healthy control. This loss of ILC3s in IBD is accompanied by an increase of ILC1s and ILC2s in inflamed samples (Geremia and Arancibia-Cárcamo, 2017; Forkel et al., 2019). They also display a notable increase in secreted IFNγ, which is associated with inflammation. Additionally, in inflamed intestinal tissue, there is an inverse relationship between the numbers of NKp44^+^ ILC3s and the accumulation of IL-17A and IFNγ^+^ T cells in human (Forkel et al., 2019). Metabolites secreted by the microbiota can influence immune responses via ILCs, the modulation of ILCs by commensals can produce helpful cytokines, and the imbalance between ILC subtypes may alter the pathogenesis of IBD (Mazmanian et al., 2005; Bernink et al., 2015).

Recently, another important role of ILC3s, its ability to perform MHC dependent functions, has been reported by various laboratories but originally from the Sonnenberg lab (Hepworth et al., 2015; Akagbosu et al., 2022; Eshleman et al., 2023). They clearly showed that ILC3s express major histocompatibility complex class II (MHCII) and such MHCII^+^ ILC3 play important roles to control dysregulated CD4 T cells during colitis. Recently, two groups showed that mice with specific deletions of MHCII on RORγt-expressing/expressed cells failed to develop microbiota-specific Tregs (considered as inducible Tregs or peripheral Tregs), thus, mice develop severe colitis (Hepworth et al., 2015; Lyu et al., 2022). Therefore, they concluded that MHCII-expressing RORγt^+^ ILC3 generate microbiota-specific regulatory T cells to establish tolerance in the gut. Although Akagbosu et al., 2022, saw similar gut inflammation phenotype using a related model (Cre-ERT2 mediated inducible deletion), they claimed that non-ILC3 RORγt^+^ antigen presenting cells as “Thetis” cells which carry combined features of mTECs and Dendritic cells. They also concluded that ILC3s were dispensable for the regulation of early-life peripheral tolerance using RORα-specific MHCII conditional deletion mice (Akagbosu et al., 2022). Two mini reviews and spotlight papers explain more detailed points (Olyha and Stephanie, 2022; Stephen-Victor and Chatila, 2022), while most ILC3s express MHCII, understanding the role of MHCII-expressing ILC3 will uncover novel features of ILC3s in terms of IBD in both mice and humans.

### What are the differences among IELs, ILCs, and T cells?

Although these three cell types share similar features, they each have their own unique markers and characteristics that we are only now starting to uncover. Firstly. IELs are closely locating in-between intestinal epithelial cells to act as a first line of defense against pathogens. IELs, unlike T cells in the lamina propria, do not need to be primed, upon encountering a pathogen. IELs will immediately begin to secrete cytokines (Zhou C. et al., 2019; Ma et al., 2021). Although until recently IELs had been underappreciated as key players in intestinal homeostasis and IBD pathogenesis, recent studies have highlighted their contributions in those contexts (Hu and Edelblum, 2017). For example, during dysbiosis, IELs have increased cytolytic potential which leads to damage to the intestinal epithelium which can exacerbate inflammatory responses (Setty et al., 2015). Similarly, it was observed that the cytokine IL-23 along with endoplasmic reticulum stress can lead to enhanced IEL lytic activity (Liu et al., 2011). Lastly, gut dysbiosis can also lead to a loss of critical regulatory IELs, which in turn then allows unwanted immune activation and inflammation (Hu and Edelblum, 2017). ILCs on the other hand, are normally located directly underneath the intestinal epithelium, a perfect position to receive signals from the epithelium, but some specialized subsets of ILCs, namely the CCR6^+^ ILC3 has been shown to home to the crypt patches to aid in crypt integrity and regeneration (Ohradanova-Repic et al., 2020). ILCs as the innate counterpart of T cells share many of the same features but importantly ILCs lack the rearranged antigen receptors that are the hallmark of conventional T cells. Since it is known that ILCs can act directly on the intestinal epithelial barrier, and can influence innate and adaptative immune responses, a comprehensive understanding on how microbiota influence ILCs is critical for future therapeutics (Ganal-Vonarburg and Duerr, 2020). It has been demonstrated that ILC3s play a critical role in IL-22 production, which leads to stimulation of repair and regenerative effects in the gut, and resistance against *Citrobacter Rodentium* infection (Guo et al., 2015). However robust study on ILC deficient mice and how specific microbiota affect them has been lacking. Finally, T cells are lymphocytes that originate as hematopoietic cells in the bone marrow and travel to the thymus to mature. For the intestines, once T cells leave the gut draining mesenteric lymph nodes (mLNs) following the antigen presentation by antigen presenting cells, they generally will reside in the underlying Lamina Propria where they partake in many different functions (Zhou C. et al., 2019; Ma et al., 2021). Many studies have focused on the role that CD4+ T helper cells (Th) play in the development of autoimmune diseases. Specifically, it was discovered that Th17 cells are strongly associated with IBD pathogenesis (Yasuda et al., 2019). During healthy intestinal conditions Th17 cells work to ensure the integrity of the intestinal epithelial barrier and communicate with other cells to produce important antimicrobial peptides (Blaschitz and Raffatellu, 2010). However, during disturbed conditions Th17 can secrete potent proinflammatory cytokines which will further enhance inflammation and IBD progression (Blaschitz and Raffatellu, 2010; Yasuda et al., 2019). Another important T cell subset is the regulatory T cell (Treg) which normally secretes immunosuppressive cytokines like IL-10 which is a key cytokine in maintaining homeostasis in the gut (Atarashi et al., 2011). It is noted that IBD patients have a distinct reduction in Tregs which might explain the increase in unwanted inflammatory responses normally seen in IBD (Mayne and Williams, 2013; Clough et al., 2020). Additionally, there is evidence that altering the precarious balance between Tregs, and T helper subsets can contribute to the pathogenesis of IBD (Mayne and Williams, 2013). A very recent paper in 2022 details how specific microbiota can influence the generation of Tregs and Th17, cells highlighting the immense influence that the microbiome has over T cell differentiation and thus what immune responses will occur (Kedmi et al., 2022). Revealing how these different cells are stimulated and the specific roles they play in IBD will enable deep analysis of the interactions between the microbiota, the intestinal epithelium, and immune cells to allow us to more precisely modulate the microbiome to enhance health of the host.

## Controlling gut inflammation with microbiota

To attenuate the inflammation in the gut during tissue damage and/or IBD, we must not only inhibit microbiome-dependent T cell dysregulation but also promote commensal-derived protective function of ILC3 and IELs. To this end, identification and characterization of specific commensal bacteria is critical to enable us to manipulate these functions. Current efforts are interested in identifying protective commensals, whether this be through their interactions with the immune cells, production of beneficial SCFAs, or inducing intestinal cell regeneration. Along these lines, it was recently revealed that there are several strains of *Clostridium* and a strain of *Faecalibacterium* that can suppress the pro inflammatory activity of NF-kB (Bajer et al., 2017; Giri et al., 2022). Moreover, *Butyrivibrio* can induce Treg derived IL-10 to maintain gut homeostasis via butyrate secretion. There are many other ways that the microbiome can modulate gut inflammation, including the stimulation of several different SCFAs such as butyrate, acetate, and propionate (Silva et al., 2020). Moreover, the gut microbiota’s ability to stimulate IL-10, TGF-B, IL-4, IL-27, and IL-35 is also associated with anti-inflammatory effects (Al Bander et al., 2020; Silva et al., 2020). Taking advantage of these beneficial commensals could be used as a treatment for IBD or other inflammatory gut diseases. Conversely identification of pathogenic commensals could be used to predict if someone may develop gut inflammation or could be used to selectively eliminated with antibiotic treatment.

Importantly, IL-23 plays a key role in the pathogenesis of autoimmune and chronic inflammatory diseases, due to IL-23R susceptibility alleles being associated with IBD. Blockade of IL-23R in human IBD animal models can often decrease gut inflammation (Buonocore et al., 2010; Parigi et al., 2022), while protective IL-22 production from ILC3s requires the stimulation of IL-23R on ILC3 (Geremia et al., 2011). On the other hand, it was reported that ILC drive innate intestinal pathology via IL-23 (Eken et al., 2014). This complexity might be due to the diverse causes of IBD development. However, to control and treat IBD, we must overcome this gap in knowledge to leverage the protective commensal-driven immune responses to improve patient outcomes. There are still many questions to fully understand the relationships among gut bacteria, gut inflammation, and immune cells in the gut (Sefik et al., 2015), but current emphasis is focusing on: (1) Will TCR specificities against gut commensals on the CD4 T cell be important factors to developing gut inflammation under disease conditions such as IL-10 KO and transfer colitis model? (2) Can commensal-inducing protective responses overcome autoimmune inflammatory signals to maintain homeostasis in the gut?

## Discussion

The microbiome in IBD patients is distinctly different than that in a healthy individual. There is overwhelming evidence that suggests that the microbiome plays a critical role in maintaining homeostatic balance to prevent dysbiosis. Studies have revealed that commensal bacteria in coordination with the innate and adaptive immune systems, IELs, and ILCs all play critical roles in these processes. Current strategies, such as the use the Fecal Microbiota Transplantation (FMT) suggests that utilizing commensals to influence the microbiome interactions with the host’s immune system to promote homeostasis and prevent dysbiosis and inflammation is a promising area of research (Lopez and Grinspan, 2016). This may include colonizing known helpful or protective bacteria to IBD patients to try and resolve dysbiosis or even supplementing known beneficial commensal-produced metabolites to a certain part of the intestine to promote intestinal barrier integrity in IBD patients. Although much of the specifics concerning which bacterial populations are helpful or harmful in these processes are still unknown. Elucidating the extensive communication between commensal bacteria with IELs and ILCs will be key in uncovering the precise mechanisms that govern intestinal homeostasis and development of IBD. Taken together the microbiome is a uniquely evolving target for diagnosis and treatment of IBD and that future studies will need to focus deeply on how particular microbes interact with the host’s tissue to promote intestinal epithelium barrier integrity, prevent inflammation, and maintain intestinal homeostasis.

## Future studies: distinguishing distinct features of IELs, ILCs, and T cells

Despite the similarity among these three cell types, many different functions have been reported. Since transcriptomic analysis became more reasonable to perform, researchers are re-considering not only these similarities but also their distinct and unique features in their expressing receptors and transcription factors. Moreover, their unique location has been considered more and more in these days. Zindl et al. nicely showed that a nonredundant function in IL-22 producing CD4 T cells and ILC3s against *Citrobacter rodentium* infection in the crypt and villi in the intestine (Zindl et al., 2022). In the near future, we will be able to analyze the host-pathogen interaction at the single cell/bacterium resolution.

## Author contributions

ZW wrote the review with supervision of TS following feedback from IC and IK. All authors contributed to the article and approved the submitted version.

## Footnotes

^1^https://www.niaid.nih.gov/diseases-conditions/autoimmune-disease-research

## References

[ref1] Abraham B. Quigley E. (2020). Antibiotics and probiotics in inflammatory bowel disease: when to use them? Frontline Gastroenterol. 11, 62–69. doi: 10.1136/flgastro-2018-101057, PMID: 31885842 PMC6914299

[ref2] Abt M. C. Lewis B. B. Caballero S. Xiong H. Carter R. A. Sušac B.. (2015). Innate immune defenses mediated by two ILC subsets are critical for protection against acute *Clostridium difficile* infection. Cell Host Microbe 18, 27–37. doi: 10.1016/j.chom.2015.06.011, PMID: 26159718 PMC4537644

[ref9001] Ahn Y. O. Weeres M. A. Neulen M. L. Choi J. Kang S. H. Heo D. S. (2015). Human group3 innate lymphoid cells express DR3 and respond to TL1A with enhanced IL-22 production and IL-2-dependent proliferation. Eur J Immunol 45, 2335–2342. 26046454 10.1002/eji.201445213 PMC4595159

[ref3] Akagbosu B. Tayyebi Z. Shibu G. Paucar Iza Y. A. Deep D. Parisotto Y. F.. (2022). Novel antigen-presenting cell imparts Treg-dependent tolerance to gut microbiota. Nature 610, 752–760. doi: 10.1038/s41586-022-05309-5, PMID: 36070798 PMC9605865

[ref5] Alam M. T. Amos G. C. A. Murphy A. R. J. Murch S. Wellington E. M. H. Arasaradnam R. P. (2020). Microbial imbalance in inflammatory bowel disease patients at different taxonomic levels. Gut Pathog. 12:1. doi: 10.1186/s13099-019-0341-6, PMID: 31911822 PMC6942256

[ref4] Al Bander Z. Nitert M. D. Mousa A. Naderpoor N. (2020). The gut microbiota and inflammation: an overview. Int. J. Environ. Res. Public Health 17:7618. doi: 10.3390/ijerph17207618, PMID: 33086688 PMC7589951

[ref6] Ananthakrishnan A. N. Kaplan G. G. Ng S. C. (2020). Changing global epidemiology of inflammatory bowel diseases: sustaining health care delivery into the 21st century. Clin. Gastroenterol. Hepatol. 18, 1252–1260. doi: 10.1016/j.cgh.2020.01.028, PMID: 32007542

[ref7] Artis D. Spits H. (2015). The biology of innate lymphoid cells. Nature 517, 293–301. doi: 10.1038/nature14189, PMID: 25592534

[ref8] Aschard H. Laville V. Tchetgen E. T. Knights D. Imhann F. Seksik P.. (2019). Genetic effects on the commensal microbiota in inflammatory bowel disease patients. PLoS Genet. 15:e1008018. doi: 10.1371/journal.pgen.1008018, PMID: 30849075 PMC6426259

[ref9] Atarashi K. Suda W. Luo C. Kawaguchi T. Motoo I. Narushima S.. (2017). Ectopic colonization of oral bacteria in the intestine drives TH1 cell induction and inflammation. Science 358, 359–365. doi: 10.1126/science.aan4526, PMID: 29051379 PMC5682622

[ref10] Atarashi K. Tanoue T. Shima T. Imaoka A. Kuwahara T. Momose Y.. (2011). Induction of colonic regulatory T cells by indigenous clostridium species. Science 331, 337–341. doi: 10.1126/science.1198469, PMID: 21205640 PMC3969237

[ref11] Bajer L. Kverka M. Kostovcik M. Macinga P. Dvorak J. Stehlikova Z.. (2017). Distinct gut microbiota profiles in patients with primary sclerosing cholangitis and ulcerative colitis. World J. Gastroenterol. 23, 4548–4558. doi: 10.3748/wjg.v23.i25.4548, PMID: 28740343 PMC5504370

[ref12] Beagley K. W. Fujihashi K. Lagoo A. S. Lagoo-Deenadaylan S. Black C. A. Murray A. M.. (1995). Differences in intraepithelial lymphocyte T cell subsets isolated from murine small versus large intestine. J. Immunol. 154, 5611–5619. doi: 10.4049/jimmunol.154.11.5611, PMID: 7751614

[ref13] Bernink J. H. Krabbendam L. Germar K. De Jong E. Gronke K. Kofoed-Nielsen M.. (2015). Interleukin-12 and -23 control plasticity of CD127(+) group 1 and group 3 innate lymphoid cells in the intestinal lamina Propria. Immunity 43, 146–160. doi: 10.1016/j.immuni.2015.06.019, PMID: 26187413

[ref14] Blaschitz C. Raffatellu M. (2010). Th17 cytokines and the gut mucosal barrier. J. Clin. Immunol. 30, 196–203. doi: 10.1007/s10875-010-9368-7, PMID: 20127275 PMC2842875

[ref15] Bousbaine D. Fisch L. I. London M. Bhagchandani P. Rezende de Castro T. B. Mimee M.. (2022). A conserved Bacteroidetes antigen induces anti-inflammatory intestinal T lymphocytes. Science 377, 660–666. doi: 10.1126/science.abg5645, PMID: 35926021 PMC9766740

[ref16] Bousso P. Lemaître F. Laouini D. Kanellopoulos J. Kourilsky P. (2000). The peripheral CD8 T cell repertoire is largely independent of the presence of intestinal flora. Int. Immunol. 12, 425–430. doi: 10.1093/intimm/12.4.425, PMID: 10744643

[ref17] Britton G. J. Contijoch E. J. Mogno I. Vennaro O. H. Llewellyn S. R. Ng R.. (2019). Microbiotas from humans with inflammatory bowel disease Alter the balance of gut Th17 and RORγt+ regulatory T cells and exacerbate colitis in mice. Immunity 50, 212–224.e4. doi: 10.1016/j.immuni.2018.12.015, PMID: 30650377 PMC6512335

[ref18] Buela K.-A. G. Omenetti S. Pizarro T. T. (2015). Crosstalk between type 3 innate lymphoid cells and the gut microbiota in inflammatory bowel disease. Curr. Opin. Gastroenterol. 31, 449–455. doi: 10.1097/MOG.0000000000000217, PMID: 26398682 PMC4682364

[ref19] Buonocore S. Ahern P. P. Uhlig H. H. Ivanov I. I. Littman D. R. Maloy K. J.. (2010). Innate lymphoid cells drive IL-23 dependent innate intestinal pathology. Nature 464, 1371–1375. doi: 10.1038/nature08949, PMID: 20393462 PMC3796764

[ref20] Camerini V. Sydora B. C. Aranda R. Nguyen C. MacLean C. McBride W.. (1993). Regional specialization of the mucosal immune system. Intraepithelial lymphocytes of the large intestine have a different phenotype and function than those of the small intestine. J. Immunol. 160, 2608–2618. doi: 10.4049/jimmunol.160.6.2608, PMID: 8345182

[ref21] Canesso M. Lemos L. Neves T. C. Marim F. M. Castro T. B. Veloso E. S.. (2017). The cytosolic sensor STING is required for intestinal homeostasis and control of inflammation. Mucosal Immunol. 11, 820–834. doi: 10.1038/mi.2017.88, PMID: 29346345

[ref22] Castellanos J. G. Woo V. Viladomiu M. Putzel G. Lima S. Diehl G. E.. (2018). Microbiota-induced TNF-like ligand 1A drives group 3 innate lymphoid cell-mediated barrier protection and intestinal T cell activation during colitis. Immunity 49, 1077–1089.e5. doi: 10.1016/j.immuni.2018.10.014, PMID: 30552020 PMC6301104

[ref23] Castleman M. J. Dillon S. M. Purba C. Cogswell A. C. McCarter M. Barker E.. (2020). Enteric bacteria induce IFNγ and Granzyme B from human colonic group 1 innate lymphoid cells. Gut Microbes 12:1667723. doi: 10.1080/19490976.2019.1667723, PMID: 31583949 PMC7524156

[ref24] Cervantes-Barragan L. Chai J. N. Tianero M. D. Di Luccia B. Ahern P. P. Merriman J.. (2017). *Lactobacillus reuteri* induces gut intraepithelial CD4+CD8αα+ T cells. Science 357, 806–810. doi: 10.1126/science.aah5825, PMID: 28775213 PMC5687812

[ref26] Chen B. Ni X. Sun R. Zeng B. Wei H. Tian Z.. (2018). Commensal bacteria-dependent CD8αβ+ T cells in the intestinal epithelium produce antimicrobial peptides. Front. Immunol. 9:1065. doi: 10.3389/fimmu.2018.01065, PMID: 29868024 PMC5964211

[ref27] Cheng J. Kalliomäki M. Heilig H. G. H. Palva A. Lähteenoja H. De Vos W. M.. (2013). Duodenal microbiota composition and mucosal homeostasis in pediatric celiac disease. BMC Gastroenterol. 13:113. doi: 10.1186/1471-230X-13-113, PMID: 23844808 PMC3716955

[ref25] Chen L. He Z. Iuga A. C. Martins Filho S. N. Faith J. J.. (2018). Diet modifies colonic microbiota and CD4+ T-cell repertoire to induce flares of colitis in mice with myeloid-cell expression of interleukin 23. Gastroenterology 155, 1177–1191.e16. doi: 10.1053/j.gastro.2018.06.034, PMID: 29909020 PMC6174107

[ref28] Chervy M. Barnich N. Denizot J. (2020). Adherent-Invasive E. coli: Update on the Lifestyle of a Troublemaker in Crohn's Disease. Int J Mol Sci 21:3734. doi: 10.3390/ijms21103734, PMID: 32466328 PMC7279240

[ref29] Chun E. Lavoie S. Fonseca-Pereira D. Bae S. Michaud M. Hoveyda H. R.. (2019). Metabolite-sensing receptor Ffar2 regulates colonic group 3 innate lymphoid cells and gut immunity. Immunity 51, 871–884.e6. doi: 10.1016/j.immuni.2019.09.014, PMID: 31628054 PMC6901086

[ref30] Clark E. Hoare C. Tanianis-Hughes J. Carlson G. L. Warhurst G. (2005). Interferon γ induces translocation of commensal *Escherichia coli* across gut epithelial cells via a lipid raft--mediated process. Gastroenterology 128, 1258–1267. doi: 10.1053/j.gastro.2005.01.046, PMID: 15887109

[ref9003] Clough J. N. Omer O. S. Tasker S. Lord G. M. and Irving P. M. (2020). Regulatory T-cell therapy in Crohn’s disease: challenges and advances. Gut 69, 942–952. 31980447 10.1136/gutjnl-2019-319850 PMC7229901

[ref31] Cohen L. J. Cho J. H. Gevers D. Chu H. (2019). Genetic factors and the intestinal microbiome guide development of microbe-based therapies for inflammatory bowel diseases. Gastroenterology 156, 2174–2189. doi: 10.1053/j.gastro.2019.03.017, PMID: 30880022 PMC6568267

[ref32] Crohn’s and Colitis Foundation. (2014). The facts about inflammatory bowel diseases. New York: Crohn’s and Colitis Foundation of America.

[ref33] Danne C. Ryzhakov G. Martínez-López M. Ilott N. E. Franchini F. Cuskin F.. (2017). A large polysaccharide produced by *Helicobacter hepaticus* induces an anti-inflammatory gene signature in macrophages. Cell Host Microbe 22, 733–745.e5. doi: 10.1016/j.chom.2017.11.002, PMID: 29241040 PMC5734933

[ref34] Das G. Augustine M. M. das J. Bottomly K. Ray P. Ray A. (2003). An important regulatory role for CD4+CD8αα T cells in the intestinal epithelial layer in the prevention of inflammatory bowel disease. Proc Natl Acad Sci U S A 100, 5324–5329. doi: 10.1073/pnas.0831037100, PMID: 12695566 PMC154344

[ref35] Diefenbach A. Gnafakis S. Shomrat O. (2020). Innate lymphoid cell-epithelial cell modules sustain intestinal homeostasis. Immunity 52, 452–463. doi: 10.1016/j.immuni.2020.02.016, PMID: 32187516

[ref36] Dieleman L. A. Arends A. Tonkonogy S. L. Goerres M. S. Craft D. W. Grenther W.. (2000). *Helicobacter hepaticus* does not induce or potentiate colitis in interleukin-10-deficient mice. Infect. Immun. 68, 5107–5113. doi: 10.1128/IAI.68.9.5107-5113.2000, PMID: 10948132 PMC101749

[ref37] Duboc H. Rajca S. Rainteau D. Benarous D. Maubert M. A. Quervain E.. (2013). Connecting dysbiosis, bile-acid dysmetabolism and gut inflammation in inflammatory bowel diseases. Gut 62, 531–539. doi: 10.1136/gutjnl-2012-302578, PMID: 22993202

[ref38] Eken A. Singh A. K. Treuting P. M. Oukka M. (2014). IL-23R+ innate lymphoid cells induce colitis via interleukin-22-dependent mechanism. Mucosal Immunol. 7, 143–154. doi: 10.1038/mi.2013.33, PMID: 23715173 PMC3834084

[ref39] Erben U. Loddenkemper C. Doerfel K. Spieckermann S. Haller D. Heimesaat M. M.. (2014). A guide to histomorphological evaluation of intestinal inflammation in mouse models. Int. J. Clin. Exp. Pathol. 7, 4557–4576. PMID: 25197329 PMC4152019

[ref40] Eshleman E. M. Shao T. Y. Woo V. Rice T. Engleman L. Didriksen B. J.. (2023). Intestinal epithelial HDAC3 and MHC class II coordinate microbiota-specific immunity. J. Clin. Invest. 133:e162190. doi: 10.1172/JCI162190, PMID: 36602872 PMC9927950

[ref41] Fakhoury M. Negrulj R. Mooranian A. Al-Salami H. (2014). Inflammatory bowel disease: clinical aspects and treatments. J. Inflamm. Res. 7, 113–120. doi: 10.2147/JIR.S65979, PMID: 25075198 PMC4106026

[ref42] Flint H. J. Scott K. P. Duncan S. H. Louis P. Forano E. (2012). Microbial degradation of complex carbohydrates in the gut. Gut Microbes 3, 289–306. doi: 10.4161/gmic.19897, PMID: 22572875 PMC3463488

[ref43] Forkel M. van Tol S. Höög C. Michaëlsson J. Almer S. Mjösberg J. (2019). Distinct alterations in the composition of mucosal innate lymphoid cells in newly diagnosed and established Crohn’s disease and ulcerative colitis. J. Crohn's Colitis 13, 67–78. doi: 10.1093/ecco-jcc/jjy119, PMID: 30496425

[ref44] Fuchs A. (2016). ILC1s in tissue inflammation and infection. Front. Immunol. 7:104. doi: 10.3389/fimmu.2016.00104, PMID: 27047491 PMC4801855

[ref45] Gaboriau-Routhiau V. Rakotobe S. Lécuyer E. Mulder I. Lan A. Bridonneau C.. (2009). The key role of segmented filamentous bacteria in the coordinated maturation of gut helper T cell responses. Immunity 31, 677–689. doi: 10.1016/j.immuni.2009.08.020, PMID: 19833089

[ref9002] Ganal‐Vonarburg S. C. Duerr C. U. (2020). The interaction of intestinal microbiota and innate lymphoid cells in health and disease throughout life. Immunology 159, 39–51. 31777064 10.1111/imm.13138 PMC6904614

[ref46] Gasaly N. De Vos P. Hermoso M. A. (2021). Impact of bacterial metabolites on gut barrier function and host immunity: a focus on bacterial metabolism and its relevance for intestinal inflammation. Front. Immunol. 12:658354. doi: 10.3389/fimmu.2021.658354, PMID: 34122415 PMC8187770

[ref47] Geremia A. Arancibia-Cárcamo C. V. (2017). Innate lymphoid cells in intestinal inflammation. Front. Immunol. 8:1296. doi: 10.3389/fimmu.2017.01296, PMID: 29081776 PMC5645495

[ref48] Geremia A. Arancibia-Cárcamo C. V. Fleming M. P. P. Rust N. Singh B. Mortensen N. J.. (2011). IL-23–responsive innate lymphoid cells are increased in inflammatory bowel disease. J. Exp. Med. 208, 1127–1133. doi: 10.1084/jem.20101712, PMID: 21576383 PMC3173242

[ref49] Giri R. Hoedt E. C. Khushi S. Salim A. A. Bergot A. S. Schreiber V.. (2022). Secreted NF-κB suppressive microbial metabolites modulate gut inflammation. Cell Rep. 39:110646. doi: 10.1016/j.celrep.2022.110646, PMID: 35417687

[ref50] Giuffrida P. Corazza G. R. Di Sabatino A. (2018). Old and new lymphocyte players in inflammatory bowel disease. Dig. Dis. Sci. 63, 277–288. doi: 10.1007/s10620-017-4892-4, PMID: 29275447

[ref51] Glassner K. L. Abraham B. P. Quigley E. M. M. (2020). The microbiome and inflammatory bowel disease. J. Allergy Clin. Immunol. 145, 16–27. doi: 10.1016/j.jaci.2019.11.003, PMID: 31910984

[ref52] Guo X. Liang Y. Zhang Y. Lasorella A. Kee B. L. Fu Y. X. (2015). Innate lymphoid cells control early colonization resistance against intestinal pathogens through ID2-dependent regulation of the microbiota. Immunity 42, 731–743. doi: 10.1016/j.immuni.2015.03.012, PMID: 25902484 PMC4725053

[ref53] Harada Y. Sujino T. Miyamoto K. Nomura E. Yoshimatsu Y. Tanemoto S.. (2022). Intracellular metabolic adaptation of intraepithelial CD4+CD8αα+ T lymphocytes. iScience 25:104021. doi: 10.1016/j.isci.2022.104021, PMID: 35313689 PMC8933710

[ref54] Helgeland L. Dissen E. Dai K. Z. Midtvedt T. Brandtzaeg P. Vaage J. T. (2004). Microbial colonization induces oligoclonal expansions of intraepithelial CD8 T cells in the gut. Eur. J. Immunol. 34, 3389–3400. doi: 10.1002/eji.200425122, PMID: 15517613

[ref55] Hepworth M. R. Fung T. C. Masur S. H. Kelsen J. R. McConnell F. M. Dubrot J.. (2015). Group 3 innate lymphoid cells mediate intestinal selection of commensal bacteria-specific CD4+ T cells. Science 348, 1031–1035. doi: 10.1126/science.aaa4812, PMID: 25908663 PMC4449822

[ref56] Hu M. D. Edelblum K. L. (2017). Sentinels at the frontline: the role of intraepithelial lymphocytes in inflammatory bowel disease. Curr. Pharmacol. Rep. 3, 321–334. doi: 10.1007/s40495-017-0105-2, PMID: 29242771 PMC5724577

[ref57] Hu M. D. Ethridge A. D. Lipstein R. Kumar S. Wang Y. Jabri B.. (2018). Epithelial IL-15 Is a Critical Regulator of γδ Intraepithelial Lymphocyte Motility within the Intestinal Mucosa. J Immunol 201, 747–756. doi: 10.4049/jimmunol.1701603, PMID: 29884699 PMC6075741

[ref58] Imaoka A. Matsumoto S. Setoyama H. Okada Y. Umesaki Y. (1996). Proliferative recruitment of intestinal intraepithelial lymphocytes after microbial colonization of germ-free mice. Eur. J. Immunol. 26, 945–948. doi: 10.1002/eji.1830260434, PMID: 8625993

[ref59] Imhann F. Vich Vila A. Bonder M. J. Fu J. Gevers D. Visschedijk M. C.. (2017). Interplay of host genetics and gut microbiota underlying the onset and clinical presentation of inflammatory bowel disease. Gut 67, 108–119. doi: 10.1136/gutjnl-2016-312135 PMC5699972 27802154

[ref60] Jang H.-M. Kim J. K. Joo M. K. Shin Y. J. Lee C. K. Kim H. J.. (2021). Transplantation of fecal microbiota from patients with inflammatory bowel disease and depression alters immune response and behavior in recipient mice. Sci. Rep. 11:20406. doi: 10.1038/s41598-021-00088-x, PMID: 34650107 PMC8516877

[ref61] Jarry A. Cerf-bensussan N. Brousse N. Selz F. Guy-grand D. (1990). Subsets of CD3+ (T cell receptor α/β or γ/δ) and CD3− lymphocytes isolated from normal human gut epithelium display phenotypical features different from their counterparts in peripheral blood. Eur. J. Immunol. 20, 1097–1103. doi: 10.1002/eji.1830200523, PMID: 2141568

[ref62] Jostins L. Ripke S. Weersma R. K. Duerr R. H. McGovern D. P. Hui K. Y.. (2012). Host-microbe interactions have shaped the genetic architecture of inflammatory bowel disease. Nature 491, 119–124. doi: 10.1038/nature11582, PMID: 23128233 PMC3491803

[ref63] Kastl A. J. Terry N. A. Wu G. D. Albenberg L. G. (2020). The structure and function of the human small intestinal microbiota: current understanding and future directions. Cell. Mol. Gastroenterol. Hepatol. 9, 33–45. doi: 10.1016/j.jcmgh.2019.07.006, PMID: 31344510 PMC6881639

[ref64] Kedmi R. Najar T. A. Mesa K. R. Grayson A. Kroehling L. Hao Y.. (2022). A RORγt+ cell instructs gut microbiota-specific Treg cell differentiation. Nature 610, 737–743. doi: 10.1038/s41586-022-05089-y, PMID: 36071167 PMC9908423

[ref65] Keubler L. M. Buettner M. Häger C. Bleich A. (2015). A multihit model: colitis lessons from the Interleukin-10–deficient mouse. Inflamm. Bowel Dis. 21, 1967–1975. doi: 10.1097/MIB.0000000000000468, PMID: 26164667 PMC4511684

[ref66] Khan I. Ullah N. Zha L. Bai Y. Khan A. Zhao T.. (2019). Alteration of gut microbiota in inflammatory bowel disease (IBD): cause or consequence? IBD treatment targeting the gut microbiome. Pathogens 8:126. doi: 10.3390/pathogens8030126, PMID: 31412603 PMC6789542

[ref67] Kho Z. Y. Lal S. K. (2018). The human gut microbiome – a potential controller of wellness and disease. Front. Microbiol. 9:1835. doi: 10.3389/fmicb.2018.01835, PMID: 30154767 PMC6102370

[ref68] Kitamoto S. Kamada N. (2022). Untangling the oral–gut axis in the pathogenesis of intestinal inflammation. Int. Immunol. 34, 485–490. doi: 10.1093/intimm/dxac027, PMID: 35716367 PMC9447993

[ref69] Kitamoto S. Nagao-Kitamoto H. Jiao Y. Gillilland M. G. Hayashi A. Imai J.. (2020). The Intermucosal connection between the mouth and gut in CommensalPathobiont-driven colitis. Cells 182, 447–462.e14. doi: 10.1016/j.cell.2020.05.048, PMID: 32758418 PMC7414097

[ref70] Kullberg M. C. Jankovic D. Feng C. G. Hue S. Gorelick P. L. McKenzie B. S.. (2006). IL-23 plays a key role in *Helicobacter hepaticus*–induced T cell–dependent colitis. J. Exp. Med. 203, 2485–2494. doi: 10.1084/jem.20061082, PMID: 17030948 PMC2118119

[ref71] Lee M. Chang E. B. (2021). Inflammatory bowel diseases and the microbiome: searching the crime scene for clues. Gastroenterology 160, 524–537. doi: 10.1053/j.gastro.2020.09.056, PMID: 33253681 PMC8098834

[ref72] Leite G. Morales W. Weitsman S. Celly S. Parodi G. Mathur R.. (2020). The duodenal microbiome is altered in small intestinal bacterial overgrowth. PLoS One 15:e0234906. doi: 10.1371/journal.pone.0234906, PMID: 32645011 PMC7347122

[ref73] Liu Z. Yadav P. K. Xu X. Su J. Chen C. Tang M.. (2011). The increased expression of IL-23 in inflammatory bowel disease promotes intraepithelial and lamina propria lymphocyte inflammatory responses and cytotoxicity. J. Leukoc. Biol. 89, 597–606. doi: 10.1189/jlb.0810456, PMID: 21227898

[ref74] Lloyd-Price J. Arze C. Ananthakrishnan A. N. Schirmer M. Avila-Pacheco J. Poon T. W.. (2019). Multi-omics of the gut microbial ecosystem in inflammatory bowel diseases. Nature 569, 655–662. doi: 10.1038/s41586-019-1237-9, PMID: 31142855 PMC6650278

[ref75] Longman R. S. Diehl G. E. Victorio D. A. Huh J. R. Galan C. Miraldi E. R.. (2014). CX3CR1+ mononuclear phagocytes support colitis-associated innate lymphoid cell production of IL-22. J. Exp. Med. 211, 1571–1583. doi: 10.1084/jem.20140678, PMID: 25024136 PMC4113938

[ref76] Longman R. S. Yang Y. Diehl G. E. Kim S. V. Littman D. R. (2013). Microbiota: host interactions in mucosal homeostasis and systemic autoimmunity. Cold Spring Harb. Symp. Quant. Biol. 78, 193–201. doi: 10.1101/sqb.2013.78.020081, PMID: 24913313 PMC4367195

[ref77] Lopez J. Grinspan A. (2016). Fecal microbiota transplantation for inflammatory bowel disease. Gastroenterol Hepatol (N Y) 12, 374–379. PMID: 27493597 PMC4971820

[ref78] Luo W. Tian L. Tan B. Shen Z. Xiao M. Wu S.. (2021). Update: innate lymphoid cells in inflammatory bowel disease. Dig. Dis. Sci. 67, 56–66. doi: 10.1007/s10620-021-06831-8, PMID: 33609209

[ref79] Lyu M. Suzuki H. Kang L. Gaspal F. Zhou W. Goc J.. (2022). ILC3s select microbiota-specific regulatory T cells to establish tolerance in the gut. Nature 610, 744–751. doi: 10.1038/s41586-022-05141-x, PMID: 36071169 PMC9613541

[ref80] Ma H. Qiu Y. Yang H. (2021). Intestinal intraepithelial lymphocytes: maintainers of intestinal immune tolerance and regulators of intestinal immunity. J. Leukoc. Biol. 109, 339–347. doi: 10.1002/JLB.3RU0220-111, PMID: 32678936 PMC7891415

[ref81] Mailhe M. Ricaboni D. Vitton V. Gonzalez J. M. Bachar D. Dubourg G.. (2018). Repertoire of the gut microbiota from stomach to colon using culturomics and next-generation sequencing. BMC Microbiol. 18:157. doi: 10.1186/s12866-018-1304-7, PMID: 30355340 PMC6201554

[ref82] Mancini N. L. Rajeev S. Jayme T. S. Wang A. Keita Å. V. Workentine M. L.. (2021). Crohn’s disease Pathobiont adherent-invasive E coli disrupts epithelial mitochondrial networks with implications for gut permeability. Cell. Mol. Gastroenterol. Hepatol. 11, 551–571. doi: 10.1016/j.jcmgh.2020.09.013, PMID: 32992049 PMC7797367

[ref83] Mariño E. Richards J. L. McLeod K. H. Stanley D. Yap Y. A. Knight J.. (2017). Gut microbial metabolites limit the frequency of autoimmune T cells and protect against type 1 diabetes. Nat. Immunol. 18, 552–562. doi: 10.1038/ni.3713, PMID: 28346408

[ref84] Martinez-Guryn K. Leone V. Chang E. B. (2019). Regional diversity of the gastrointestinal microbiome. Cell Host Microbe 26, 314–324. doi: 10.1016/j.chom.2019.08.011, PMID: 31513770 PMC6750279

[ref85] Masopust D. Choo D. Vezys V. Wherry E. J. Duraiswamy J. Akondy R.. (2010). Dynamic T cell migration program provides resident memory within intestinal epithelium. J. Exp. Med. 207, 553–564. doi: 10.1084/jem.20090858, PMID: 20156972 PMC2839151

[ref9004] Mayne C. G. Williams C. B. (2013). Induced and natural regulatory T cells in the development of inflammatory bowel disease. Inflamm Bowel Dis 19, 1772–1788. 23656897 10.1097/MIB.0b013e318281f5a3 PMC3690174

[ref86] Mazmanian S. K. Liu C. H. Tzianabos A. O. Kasper D. L. (2005). An immunomodulatory molecule of symbiotic bacteria directs maturation of the host immune system. Cells 122, 107–118. doi: 10.1016/j.cell.2005.05.007, PMID: 16009137

[ref87] Mentella M. C. Scaldaferri F. Pizzoferrato M. Gasbarrini A. Miggiano G. A. D. (2020). Nutrition, IBD and gut microbiota: a review. Nutrients 12:944. doi: 10.3390/nu12040944, PMID: 32235316 PMC7230231

[ref88] Michaudel C. Sokol H. (2020). The gut microbiota at the Service of Immunometabolism. Cell Metab. 32, 514–523. doi: 10.1016/j.cmet.2020.09.004, PMID: 32946809

[ref89] Miljković Đ. Jevtić B. Stojanović I. Dimitrijević M. (2021). ILC3, a central innate immune component of the gut-brain Axis in multiple sclerosis. Front. Immunol. 12:1025. doi: 10.3389/fimmu.2021.657622 PMC8071931 33912185

[ref90] Mizoguchi A. Yano A. Himuro H. Ezaki Y. Sadanaga T. Mizoguchi E. (2018). Clinical importance of IL-22 cascade in IBD. J. Gastroenterol. 53, 465–474. doi: 10.1007/s00535-017-1401-7, PMID: 29075900 PMC5866830

[ref91] Morrissey P. J. Charrier K. Horovitz D. A. Fletcher F. A. Watson J. D. (1995). Analysis of the intra-epithelial lymphocyte compartment in SCID mice that received co-isogenic CD4+ T cells. Evidence that mature post-thymic CD4+ T cells can be induced to express CD8 alpha in vivo. J. Immunol. 154, 2678–2686. doi: 10.4049/jimmunol.154.6.2678, PMID: 7876540

[ref92] Mortha A. Burrows K. (2018). Cytokine networks between innate lymphoid cells and myeloid cells. Front. Immunol. 9:191. doi: 10.3389/fimmu.2018.00191, PMID: 29467768 PMC5808287

[ref93] Mortha A. Chudnovskiy A. Hashimoto D. Bogunovic M. Spencer S. P. Belkaid Y.. (2014). Microbiota-dependent crosstalk between macrophages and ILC3 promotes intestinal homeostasis. Science 343:1249288. doi: 10.1126/science.1249288, PMID: 24625929 PMC4291125

[ref94] Nardone G. Compare D. (2015). The human gastric microbiota: is it time to rethink the pathogenesis of stomach diseases? United European Gastroenterol J 3, 255–260. doi: 10.1177/2050640614566846, PMID: 26137299 PMC4480535

[ref95] Nielsen M. M. Witherden D. A. Havran W. L. (2017). γδ T cells in homeostasis and host defence of epithelial barrier tissues. Nat. Rev. Immunol. 17, 733–745. doi: 10.1038/nri.2017.101, PMID: 28920588 PMC5771804

[ref96] Nitzan O. Elias M. Peretz A. Saliba W. (2016). Role of antibiotics for treatment of inflammatory bowel disease. World J. Gastroenterol. 22, 1078–1087. doi: 10.3748/wjg.v22.i3.1078, PMID: 26811648 PMC4716021

[ref97] Ohradanova-Repic A. Boes M. Stockinger H. (2020). Role of metabolism in regulating immune cell fate decisions. Lausanne, Switzerland: Frontiers Media SA. 10.3389/fimmu.2020.00527 PMC7105706 32265941

[ref98] Olfatifar M. Zali M. R. Pourhoseingholi M. A. Balaii H. Ghavami S. B. Ivanchuk M.. (2021). The emerging epidemic of inflammatory bowel disease in Asia and Iran by 2035: a modeling study. BMC Gastroenterol. 21:204. doi: 10.1186/s12876-021-01745-1, PMID: 33957874 PMC8101120

[ref99] Olyha S. J. Stephanie C. (2022). Eisenbarth. A new tolerogenic cell RORs onto the scene. Science Immunology 7:eadf0767. doi: 10.1126/sciimmunol.adf0767, PMID: 36206352

[ref100] Ostanin D. V. Bao J. Koboziev I. Gray L. Robinson-Jackson S. A. Kosloski-Davidson M.. (2009). T cell transfer model of chronic colitis: concepts, considerations, and tricks of the trade. Am. J. Physiol. Gastrointest. Liver Physiol. 296, G135–G146. doi: 10.1152/ajpgi.90462.2008, PMID: 19033538 PMC2643911

[ref101] Pabst O. Slack E. (2020). IgA and the intestinal microbiota: the importance of being specific. Mucosal Immunol. 13, 12–21. doi: 10.1038/s41385-019-0227-4, PMID: 31740744 PMC6914667

[ref102] Palm N. W. de Zoete M. R. Cullen T. W. Barry N. A. Stefanowski J. Hao L.. (2014). Immunoglobulin a coating identifies Colitogenic bacteria in inflammatory bowel disease. Cells 158, 1000–1010. doi: 10.1016/j.cell.2014.08.006, PMID: 25171403 PMC4174347

[ref103] Panda S. K. Colonna M. (2019). Innate lymphoid cells in mucosal immunity. Front. Immunol. 10:861. doi: 10.3389/fimmu.2019.00861, PMID: 31134050 PMC6515929

[ref104] Parigi T. L. Iacucci M. Ghosh S. (2022). Blockade of IL-23: what is in the pipeline? J. Crohns Colitis 16, ii64–ii72. doi: 10.1093/ecco-jcc/jjab185, PMID: 35553666 PMC9097679

[ref105] Pickard J. M. Zeng M. Y. Caruso R. Núñez G. (2017). Gut microbiota: role in pathogen colonization, immune responses, and inflammatory disease. Immunol. Rev. 279, 70–89. doi: 10.1111/imr.12567, PMID: 28856738 PMC5657496

[ref106] Pickert G. Neufert C. Leppkes M. Zheng Y. Wittkopf N. Warntjen M.. (2009). STAT3 links IL-22 signaling in intestinal epithelial cells to mucosal wound healing. J. Exp. Med., –1472. doi: 10.1084/jem.20082683, PMID: 19564350 PMC2715097

[ref107] Poholek C. H. Dulson S. J. Zajac A. J. Harrington L. E. (2019). Interleukin-21 controls ILC3 cytokine production and promotes a protective phenotype in a mouse model of colitis. Immunohorizons 3, 194–202. doi: 10.4049/immunohorizons.1900005, PMID: 31356165 PMC6788290

[ref108] Powell N. Walker A. W. Stolarczyk E. Canavan J. B. Gökmen M. R. Marks E.. (2012). The transcription factor T-bet regulates intestinal inflammation mediated by interleukin-7 receptor+ innate lymphoid cells. Immunity 37, 674–684. doi: 10.1016/j.immuni.2012.09.008, PMID: 23063332 PMC3540260

[ref109] Powrie F. Coffman R. L. Correa-Oliveira R. (1994). Transfer of CD4+ T cells to C.B-17 SCID mice: a model to study Th1 and Th2 cell differentiation and regulation in vivo. Res. Immunol. 145, 347–353. doi: 10.1016/S0923-2494(94)80198-3, PMID: 7701113

[ref110] Pu Q. Lin P. Gao P. Wang Z. Guo K. Qin S.. (2021). Gut microbiota regulate gut-lung Axis inflammatory responses by mediating ILC2 compartmental migration. J. Immunol. 207, 257–267. doi: 10.4049/jimmunol.2001304, PMID: 34135060 PMC8674377

[ref111] Qiu J. Heller J. J. Guo X. Chen Z. M. E. Fish K. Fu Y. X.. (2012). The aryl hydrocarbon receptor regulates gut immunity through modulation of innate lymphoid cells. Immunity 36, 92–104. doi: 10.1016/j.immuni.2011.11.011, PMID: 22177117 PMC3268875

[ref112] Reece P. Gauvreau G. M. Sehmi R. Denburg J. A. (2014). IL-4 and IL-13 differentially regulate TLR-induced eosinophil-basophil differentiation of cord blood CD34+ progenitor cells. PLoS One 9:e100734. doi: 10.1371/journal.pone.0100734, PMID: 24971469 PMC4074087

[ref113] Reinoso Webb C. den Bakker H. Koboziev I. Jones-Hall Y. Rao Kottapalli K. Ostanin D.. (2018). Differential susceptibility to T cell-induced colitis in mice: role of the intestinal microbiota. Inflamm. Bowel Dis. 24, 361–379. doi: 10.1093/ibd/izx014, PMID: 29361089 PMC6176899

[ref114] Romera-Hernández M. Aparicio-Domingo P. Papazian N. Karrich J. J. Cornelissen F. Hoogenboezem R. M.. (2020). Yap1-driven intestinal repair is controlled by group 3 innate lymphoid cells. Cell Rep. 30, 37–45.e3. doi: 10.1016/j.celrep.2019.11.115, PMID: 31914395

[ref115] Saez A. Gomez-Bris R. Herrero-Fernandez B. Mingorance C. Rius C. Gonzalez-Granado J. M. (2021). Innate lymphoid cells in intestinal homeostasis and inflammatory bowel disease. Int. J. Mol. Sci. 22:7618. doi: 10.3390/ijms22147618, PMID: 34299236 PMC8307624

[ref116] Sano T. Huang W. Hall J. A. Yang Y. Chen A. Gavzy S. J.. (2015). An IL-23R/IL-22 circuit regulates epithelial serum amyloid a to promote local effector Th17 responses. Cells 163, 381–393. doi: 10.1016/j.cell.2015.08.061, PMID: 26411290 PMC4621768

[ref117] Sarrabayrouse G. Bossard C. Chauvin J. M. Jarry A. Meurette G. Quévrain E.. (2014). CD4CD8αα lymphocytes, a novel human regulatory T cell subset induced by colonic bacteria and deficient in patients with inflammatory bowel disease. PLoS Biol. 12:e1001833. doi: 10.1371/journal.pbio.1001833, PMID: 24714093 PMC3979654

[ref118] Sartor R. B. (1997). The influence of normal microbial flora on the development of chronic mucosal inflammation. Res. Immunol. 148, 567–576. doi: 10.1016/S0923-2494(98)80151-X, PMID: 9588836

[ref119] Schaubeck M. Clavel T. Calasan J. Lagkouvardos I. Haange S. B. Jehmlich N.. (2016). Dysbiotic gut microbiota causes transmissible Crohn’s disease-like ileitis independent of failure in antimicrobial defence. Gut 65, 225–237. doi: 10.1136/gutjnl-2015-309333, PMID: 25887379 PMC4752651

[ref120] Schirmer M. Franzosa E. A. Lloyd-Price J. McIver L. J. Schwager R. Poon T. W.. (2018). Dynamics of metatranscription in the inflammatory bowel disease gut microbiome. Nat. Microbiol. 3, 337–346. doi: 10.1038/s41564-017-0089-z, PMID: 29311644 PMC6131705

[ref121] Sefik E. Geva-Zatorsky N. Oh S. Konnikova L. Zemmour D. McGuire A. M.. (2015). Individual intestinal symbionts induce a distinct population of RORγ+ regulatory T cells. Science 349, 993–997. doi: 10.1126/science.aaa9420, PMID: 26272906 PMC4700932

[ref122] Sellon R. K. Tonkonogy S. Schultz M. Dieleman L. A. Grenther W. Balish E.. (1998). Resident enteric bacteria are necessary for development of spontaneous colitis and immune system activation in Interleukin-10-deficient mice. Infect. Immun. 66, 5224–5231. doi: 10.1128/IAI.66.11.5224-5231.1998, PMID: 9784526 PMC108652

[ref123] Sepahi A. Liu Q. Friesen L. Kim C. H. (2021). Dietary fiber metabolites regulate innate lymphoid cell responses. Mucosal Immunol. 14, 317–330. doi: 10.1038/s41385-020-0312-8, PMID: 32541842 PMC7736174

[ref124] Sepehri S. Kotlowski R. Bernstein C. N. Krause D. O. (2007). Microbial diversity of inflamed and noninflamed gut biopsy tissues in inflammatory bowel disease. Inflamm. Bowel Dis. 13, 675–683. doi: 10.1002/ibd.20101, PMID: 17262808

[ref125] Setty M. Discepolo V. Abadie V. Kamhawi S. Mayassi T. Kent A.. (2015). Distinct and synergistic contributions of epithelial stress and adaptive immunity to functions of intraepithelial killer cells and active celiac disease. Gastroenterology 149, 681–691.e10. doi: 10.1053/j.gastro.2015.05.013, PMID: 26001928 PMC4550536

[ref126] Silva Y. P. Bernardi A. Frozza R. L. (2020). The role of short-chain fatty acids from gut microbiota in gut-brain communication. Front. Endocrinol. 11:25. doi: 10.3389/fendo.2020.00025, PMID: 32082260 PMC7005631

[ref127] Simpson S. J. Holländer G. A. Mizoguchi E. Allen D. Bhan A. K. Wang B.. (1997). Expression of pro-inflammatory cytokines by TCRαβ+ T and TCRγδ+ T cells in an experimental model of colitis. Eur. J. Immunol. 27, 17–25. doi: 10.1002/eji.1830270104, PMID: 9021993

[ref128] Somineni H. K. Kugathasan S. (2019). The microbiome in patients with inflammatory diseases. Clin. Gastroenterol. Hepatol. 17, 243–255. doi: 10.1016/j.cgh.2018.08.078, PMID: 30196163

[ref129] Song X. Zhang H. Zhang Y. Goh B. Bao B. Mello S. S.. (2023). Gut microbial fatty acid isomerization modulates intraepithelial T cells. Nature 619, 837–843. doi: 10.1038/s41586-023-06265-4, PMID: 37380774

[ref130] Sonnenberg G. F. Hepworth M. R. (2019). Functional interactions between innate lymphoid cells and adaptive immunity. Nat. Rev. Immunol. 19, 599–613. doi: 10.1038/s41577-019-0194-8, PMID: 31350531 PMC6982279

[ref131] Sonnenberg G. F. Monticelli L. A. Elloso M. M. Fouser L. A. Artis D. (2011). CD4(+) lymphoid tissue-inducer cells promote innate immunity in the gut. Immunity 34, 122–134. doi: 10.1016/j.immuni.2010.12.009, PMID: 21194981 PMC3035987

[ref132] Stephen-Victor E. Chatila T. A. (2022). An embarrassment of riches: RORγt+ antigen-presenting cells in peripheral tolerance. Immunity 55, 1978–1980. doi: 10.1016/j.immuni.2022.10.009, PMID: 36351372 PMC10069454

[ref133] Sujino T. London M. Hoytema van Konijnenburg D. Rendon T. Buch T. Silva H. M.. (2016). Tissue adaptation of regulatory and intraepithelial CD4+ T cells controls gut inflammation. Science 352, 1581–1586. doi: 10.1126/science.aaf3892, PMID: 27256884 PMC4968079

[ref134] Sundin O. H. Mendoza-Ladd A. Zeng M. Diaz-Arévalo D. Morales E. Fagan B. M.. (2017). The human jejunum has an endogenous microbiota that differs from those in the oral cavity and colon. BMC Microbiol. 17:160. doi: 10.1186/s12866-017-1059-6, PMID: 28716079 PMC5513040

[ref135] Takatsu K. (2011). Interleukin-5 and IL-5 receptor in health and diseases. Proc. Jpn. Acad. Ser. B Phys. Biol. Sci. 87, 463–485. doi: 10.2183/pjab.87.463, PMID: 21986312 PMC3313690

[ref136] Tamura A. Soga H. Yaguchi K. Yamagishi M. Toyota T. Sato J.. (2003). Distribution of two types of lymphocytes (intraepithelial and lamina-propria-associated) in the murine small intestine. Cell Tissue Res. 313, 47–53. doi: 10.1007/s00441-003-0706-4, PMID: 12827490

[ref137] Thursby E. Juge N. (2017). Introduction to the human gut microbiota. Biochem. J. 474, 1823–1836. doi: 10.1042/BCJ20160510, PMID: 28512250 PMC5433529

[ref138] Umesaki Y. Setoyama H. Matsumoto S. Imaoka A. Itoh K. (1999). Differential roles of segmented filamentous bacteria and clostridia in development of the intestinal immune system. Infect. Immun. 67, 3504–3511. doi: 10.1128/IAI.67.7.3504-3511.1999, PMID: 10377132 PMC116537

[ref140] Van De Pavert S. A. (2021). Lymphoid tissue inducer (LTi) cell ontogeny and functioning in embryo and adult. Biom. J. 44, 123–132. doi: 10.1016/j.bj.2020.12.003, PMID: 33849806 PMC8178546

[ref139] Van Kaer L. Olivares-Villagómez D. (2018). Development, homeostasis, and functions of intestinal intraepithelial lymphocytes. J.I. 200, 2235–2244. doi: 10.4049/jimmunol.1701704, PMID: 29555677 PMC5863587

[ref141] Viladomiu M. Kivolowitz C. Abdulhamid A. Dogan B. Victorio D. Castellanos J. G.. (2017). IgA-coated *E. coli* enriched in Crohn’s disease spondyloarthritis promote TH17-dependent inflammation. Sci. Transl. Med. 9:eaaf9655. doi: 10.1126/scitranslmed.aaf9655, PMID: 28179509 PMC6159892

[ref142] Villmones H. C. Haug E. S. Ulvestad E. Grude N. Stenstad T. Halland A.. (2018). Species level description of the human Ileal bacterial microbiota. Sci. Rep. 8:4736. doi: 10.1038/s41598-018-23198-5, PMID: 29549283 PMC5856834

[ref143] Vivier E. Artis D. Colonna M. Diefenbach A. Di Santo J. P. Eberl G.. (2018). Innate lymphoid cells: 10 years on. Cells 174, 1054–1066. doi: 10.1016/j.cell.2018.07.017, PMID: 30142344

[ref144] Vuik F. Dicksved J. Lam S. Y. Fuhler G. M. van der Laan L. van de Winkel A.. (2019). Composition of the mucosa-associated microbiota along the entire gastrointestinal tract of human individuals. United European Gastroenterol J 7, 897–907. doi: 10.1177/2050640619852255, PMID: 31428414 PMC6683645

[ref145] Wang M. Ahrné S. Jeppsson B. Molin G. (2005). Comparison of bacterial diversity along the human intestinal tract by direct cloning and sequencing of 16S rRNA genes. FEMS Microbiol. Ecol. 54, 219–231. doi: 10.1016/j.femsec.2005.03.012, PMID: 16332321

[ref146] Weingarden A. R. Vaughn B. P. (2017). Intestinal microbiota, fecal microbiota transplantation, and inflammatory bowel disease. Gut Microbes 8, 238–252. doi: 10.1080/19490976.2017.1290757, PMID: 28609251 PMC5479396

[ref147] Whary M. T. Taylor N. S. Feng Y. Ge Z. Muthupalani S. Versalovic J.. (2011). *Lactobacillus reuteri* promotes *Helicobacter hepaticus*-associated typhlocolitis in gnotobiotic B6.129P2-IL-10 (tm1Cgn) (IL-10 (−/−)) mice. Immunology 133, 165–178. doi: 10.1111/j.1365-2567.2011.03423.x, PMID: 21426337 PMC3088979

[ref148] Willinger T. (2019). Metabolic control of innate lymphoid cell migration. Front. Immunol. 10:2010. doi: 10.3389/fimmu.2019.02010, PMID: 31507605 PMC6713999

[ref149] Wu W. M. Yang Y. S. Peng L. H. (2014). Microbiota in the stomach: new insights. J. Dig. Dis. 15, 54–61. doi: 10.1111/1751-2980.12116, PMID: 24245792

[ref150] Xuan X. Zhang L. Tian C. Wu T. Ye H. Cao J.. (2021). Interleukin-22 and connective tissue diseases: emerging role in pathogenesis and therapy. Cell Biosci. 11:2. doi: 10.1186/s13578-020-00504-1, PMID: 33407883 PMC7788945

[ref151] Yang H. Antony P. A. Wildhaber B. E. Teitelbaum D. H. (2004). Intestinal intraepithelial lymphocyte γδ-T cell-derived keratinocyte growth factor modulates epithelial growth in the mouse. J. Immunol. 172, 4151–4158. doi: 10.4049/jimmunol.172.7.4151, PMID: 15034027

[ref152] Yang I. Nell S. Suerbaum S. (2013). Survival in hostile territory: the microbiota of the stomach. FEMS Microbiol. Rev. 37, 736–761. doi: 10.1111/1574-6976.12027, PMID: 23790154

[ref153] Yap Y. A. Mariño E. (2018). An insight into the intestinal web of mucosal immunity, microbiota, and diet in inflammation. Front. Immunol. 9:2617. doi: 10.3389/fimmu.2018.02617, PMID: 30532751 PMC6266996

[ref154] Yasuda K. Takeuchi Y. Hirota K. (2019). The pathogenicity of Th17 cells in autoimmune diseases. Semin. Immunopathol. 41, 283–297. doi: 10.1007/s00281-019-00733-8, PMID: 30891627

[ref155] Zhang Y. Huang B. (2017). The development and diversity of ILCs, NK cells and their relevance in health and diseases. Adv. Exp. Med. Biol. 1024, 225–244. doi: 10.1007/978-981-10-5987-2_11, PMID: 28921473

[ref156] Zhao M. Gönczi L. Lakatos P. L. Burisch J. (2021). The burden of inflammatory bowel disease in Europe in 2020. J. Crohn's Colitis 15, 1573–1587. doi: 10.1093/ecco-jcc/jjab029, PMID: 33582812

[ref158] Zhou C. Qiu Y. Yang H. (2019). CD4CD8αα IELs: they have something to say. Front. Immunol. 10:2269. doi: 10.3389/fimmu.2019.02269, PMID: 31649659 PMC6794356

[ref157] Zhou L. Chu C. Teng F. Bessman N. J. Goc J. Santosa E. K.. (2019). Innate lymphoid cells support regulatory T cells in the intestine through interleukin-2. Nature 568, 405–409. doi: 10.1038/s41586-019-1082-x, PMID: 30944470 PMC6481643

[ref159] Zhou W. Sonnenberg G. F. (2020). Activation and suppression of group 3 innate lymphoid cells in the gut. Trends Immunol. 41, 721–733. doi: 10.1016/j.it.2020.06.009, PMID: 32646594 PMC7395873

[ref160] Zhu L. Zhu C. Cao S. Zhang Q. (2021). *Helicobacter hepaticus* induce colitis in male IL-10−/− mice dependent by Cytolethal distending toxin B and via the activation of Jak/stat signaling pathway. Front. Cell. Infect. Microbiol. 11:616218. doi: 10.3389/fcimb.2021.616218, PMID: 33777833 PMC7994616

[ref161] Zindl C. L. Witte S. J. Laufer V. A. Gao M. Yue Z. Janowski K. M.. (2022). A nonredundant role for T cell-derived interleukin 22 in antibacterial defense of colonic crypts. Immunity 55, 494–511.e11. doi: 10.1016/j.immuni.2022.02.003, PMID: 35263568 PMC9126440

